# Corynebacterium amycolatum: An Emerging Pathogen in Chronic Otitis Media

**DOI:** 10.7759/cureus.109362

**Published:** 2026-05-21

**Authors:** Graciela Ibarra-Armenta, Melissa Maribel Angulo-Altamirano, Alan Yovani Valle-Obeso, Evangelina Sotolongo-Barroso, Erika Celis-Aguilar, Jesus Marlen Martinez-Rivera

**Affiliations:** 1 Otolaryngology - Head and Neck Surgery, Hospital Civil de Culiacán, Universidad Autónoma de Sinaloa, Culiacan, MEX; 2 Microbiology, Laboratorio Delia Barraza, Culiacan, MEX

**Keywords:** antibiogram, antibiotics, cholesteatoma, corynebacterium amycolatum, otitis

## Abstract

Background: *Corynebacterium amycolatum* has traditionally been considered a commensal organism of the skin and mucosa; however, recent reports suggest its emerging role as a true pathogen in chronic otitis media (COM). Understanding its microbiological characteristics and resistance profile is essential for guiding effective antimicrobial therapy.

Objectives: To describe the characteristics of *C. amycolatum* infection in patients with COM, evaluating its antibiotic susceptibility and resistance patterns.

Materials and methods: A retrospective study was conducted on patients diagnosed with COM who had positive cultures for *C. amycolatum* between June 2022 and February 2025 at a secondary care center. Microbiological results, including susceptibility and resistance profiles, were analyzed using descriptive statistics.

Results: During the study period, a total of 93 ear cultures were analyzed, of which 18 were positive for *Corynebacterium* species, including 12 cases of *C. amycolatum*. Eight had a single positive culture, while four had multiple positive cultures. *C. amycolatum* coexisted with other bacteria in eight cultures, 62% of which were coagulase-negative *Staphylococcus*. High resistance rates were observed for trimethoprim/sulfamethoxazole (100%), clindamycin (90%), levofloxacin (86.6%), and gentamicin (89%), while 100% susceptibility was noted for cefotaxime, cefepime, vancomycin, tetracycline, tigecycline, and linezolid.

Conclusions: *C. amycolatum* is an emerging pathogen in COM, particularly in patients with cholesteatoma. There is also significant resistance to commonly used antibiotics, such as quinolones and penicillins. These findings highlight the need for accurate microbiological diagnosis and the implementation of targeted therapies to optimize treatment and prevent antimicrobial resistance.

## Introduction

Chronic otitis media (COM) is defined as a chronic inflammatory process of the mucoperiosteum with an insidious onset, slow progression, and a duration of more than three months. It affects the structures of the middle ear cavity, mastoid air cells, and the Eustachian tube [1]. Despite therapeutic advances, COM remains a significant global health concern due to its chronicity, complications, and evolving patterns of microbial resistance.

The microbiology of COM involves the presence of various microorganisms, including fungi and bacteria, within the middle ear [2]. In a prospective study from Western Rajasthan, Gupta and Kumbhat reported that the most common organisms recovered in COM were *Pseudomonas* spp. (58%) and *Staphylococcus aureus* (25%), with fungal isolates accounting for about 8% of cases [3].

However, recent studies have highlighted the emergence of less-recognized pathogens such as *Corynebacterium amycolatum*, which are often dismissed as contaminants due to their commensal presence on the skin and mucous membranes [4]. Misidentification or under-recognition of these organisms may contribute to inadequate treatment and persistent infection.

*C. amycolatum* is a non-lipophilic, fermentative corynebacterium first described in 1988. It is one of the most frequently isolated nondiphtherial corynebacteria, with a distinctive growth pattern. However, it is often difficult to distinguish between infection, colonization, and contamination, as it is a commensal organism of the skin and mucous membranes [4-6]. A causal role is more likely when polymorphonuclear leukocytes are observed in the clinical specimen along with gram-positive bacilli, especially in the absence of any other pathogens [7]. It is a biofilm-forming bacterium with strong adherence capacity, which protects it from antibiotics and the immune response. Conversely, immune activation in response to its biofilm can cause collateral tissue damage due to the release of phagocytic enzymes, free radicals, and the formation of immune complexes [8,9].

There are a few studies describing human ear infections caused by *C. amycolatum*. A 2015 study presented 12 cases of ear infections caused by *C. amycolatum* diagnosed using the VITEK 2 system and PCR. Of the 12 patients, seven had pure growth of *C. amycolatum*, while the remaining had growth of other organisms; three samples showed *C. amycolatum* along with *Pseudomonas aeruginosa*, and two had *C. amycolatum* with *S. aureus* [7].

Antibiotic resistance patterns of the bacteria causing otitis media vary by geographic region and must be the cornerstone for establishing therapeutic recommendations. Therefore, it is essential to understand local antibiotic resistance patterns [10,11]. 

Antimicrobial resistance among *Corynebacterium* species is increasingly reported. Studies have shown high resistance to β-lactams, macrolides, fluoroquinolones, and clindamycin, while maintaining susceptibility to glycopeptides and oxazolidinones such as vancomycin and linezolid [7,9,12].

In a retrospective study conducted by the Department of Medical Microbiology at the Jiménez Díaz Foundation, the resistance rates were 38.9% for ampicillin, 44.4% for cefazolin, and 94.4% for erythromycin, clindamycin, and ciprofloxacin, while only 11.1% showed resistance to rifampicin [5]. In a case series, 100% susceptibility to vancomycin and 66.6% resistance to ceftriaxone were reported [6,7]. Although vancomycin remains the most effective therapeutic option, recent reports highlight potential adverse reactions such as drug-induced hypersensitivity and Drug Reaction With Eosinophilia and Systemic Symptoms (DRESS) syndrome, particularly with prolonged or high-dose therapy [13,14]. This underscores the importance of continuous surveillance of antibiotic susceptibility and careful therapeutic selection.

Therapeutic approaches for *C. amycolatum* and other biofilm-forming bacteria must be based on understanding the effects of antibiotics on their structure. Inadequate treatment may lead to rapid bacterial growth, detachment, and dissemination into the bloodstream and surrounding tissues [15].

The objective of this study is to describe *C. amycolatum* infection in patients with COM, as well as its antibiotic susceptibility and resistance patterns.

## Materials and methods

A retrospective observational study was conducted using the electronic medical records of patients diagnosed with COM at a secondary care center between June 2022 and January 2025. Previous wording of the methodology unintentionally suggested that cases with co-isolates had been excluded; this has now been corrected for accuracy. The study included all patients with middle ear cultures positive for *C. amycolatum*, either as a single isolate or in polymicrobial cultures. Cases were identified through microbiology laboratory reports and clinical documentation.

Inclusion criteria were patients diagnosed with COM, availability of microbiological culture results reporting *C. amycolatum*, whether isolated alone or in combination with other microorganisms, and complete clinical records, including antibiotic susceptibility results and treatment information.

Exclusion criteria were patients without a confirmed COM diagnosis, no isolation of *C. amycolatum* in microbiological culture, and incomplete medical or microbiological data.

Although some samples yielded mixed bacterial growth, inclusion was justified when *C. amycolatum* was considered a clinically significant isolate based on consistent growth in repeated cultures and correlation with active otorrhea and inflammation on otoscopic examination. These criteria differentiated true infection from colonization.

Samples for culture were obtained using sterile transport swabs (Transystem™ STUART W/O CH) and processed in an external microbiology laboratory. Culture media included blood agar, chocolate agar, MacConkey agar, and Sabouraud agar. Plates were incubated at 37°C with CO₂ for approximately 72 hours for bacteria and up to 21 days for fungi. All procedures were performed under biosafety level II conditions using Densichek Plus and semiautomatic pipettes.

The VITEK® 2 system (bioMérieux, Marcy-l'Étoile, France) is an automated antimicrobial susceptibility testing (AST) system that includes instruments, software, and disposable reagent cards. It received FDA approval in 1998-1999 for AST of bacteria from pure colonies [16].

Microorganism identification is carried out using specific identification cards, such as ID-GPC for Gram-positive cocci, ID-GNB for Gram-negative bacilli, and ID-YST for yeasts. These cards contain multiple biochemical tests that enable rapid species-level identification [17-19]. In addition, the system performs AST to evaluate microorganism susceptibility to various antibiotics using cards with preset antibiotic concentrations [18].

The system is designed to optimize laboratory workflow by reducing manual handling time, providing rapid results (6-8 hours), and using updated databases and algorithms to ensure accurate identification and susceptibility profiles [20,21].

The data collected included whether the patient had cholesteatoma, relevant medical and surgical history, culture results, and antibiotic treatment (levofloxacin, moxifloxacin, cefotaxime, ceftriaxone, cefepime, penicillin, ampicillin, benzylpenicillin, gentamicin, erythromycin, clindamycin, vancomycin, tetracycline, tigecycline, trimethoprim/sulfamethoxazole, and linezolid).

This study was conducted in accordance with institutional ethical standards. Due to its retrospective design and the use of previously collected, deidentified clinical and microbiological data, the Institutional Ethics Committee determined that formal ethical approval and an approval number were not required and granted an exemption from review. The requirement for informed patient consent was waived. The study did not involve direct patient contact or intervention, and patient confidentiality was strictly maintained throughout data collection and analysis.

## Results

During the study period, a total of 93 ear cultures from patients with COM were analyzed. *Corynebacterium* species were isolated in 18 cultures, representing 19.3% of all cultures, of which *C. amycolatum* accounted for the majority of identified isolates.

Twelve patients with COM who had one or more positive cultures for *C. amycolatum* were included. The mean age of the patients was 44 years (range: 2-84 years); 58.3% (n=7) were female, and 41.6% (n=5) were male. Reported comorbidities included diabetes mellitus in 16.6% (n=2), arterial hypertension in 50% (n=6), overweight or obesity in 58.3% (n=7), and cholesteatoma in 92% (n=11).

Eight patients had a single positive culture, while four patients had more than one positive culture (Table 1 and Table 2). Among the 12 positive cultures, eight were polymicrobial, with *C. amycolatum* coexisting with other bacteria. The most frequent coinfecting organism was coagulase-negative Staphylococcus (62%), followed by *Proteus mirabilis* and *Enterococcus* species.

**Table 1:** Initial cultures of patients with Corynebacterium amycolatum It presents the initial cultures of all patients, the presence of cholesteatoma, isolation of *Corynebacterium amycolatum*, and coinfecting bacteria.

Patient ID	Presence of cholesteatoma	Isolated microorganism	Co-infection microorganism
1	Yes	Corynebacterium amycolatum	Coagulase-negative* Staphylococci*
2	Yes	Corynebacterium amycolatum	Coagulase-negative* Staphylococci, Candida parapsilosis*
3	Yes	Corynebacterium amycolatum	None
4	Yes	Corynebacterium amycolatum	None
5	Yes	Corynebacterium amycolatum	None
6	Yes	Corynebacterium amycolatum	Achromobacter xylosoxidans
7	Yes	Corynebacterium amycolatum	Coagulase-negative* Staphylococci, Aspergillus flavus*
8	Yes	Corynebacterium amycolatum	Proteus mirabilis
9	Yes	Corynebacterium amycolatum	Coagulase-negative* Staphylococci*
10	Yes	Corynebacterium amycolatum	Enterococcus faecalis
11	No	Corynebacterium amycolatum	None
12	Yes	Corynebacterium amycolatum	Coagulase-negative *Staphylococci*

**Table 2:** Repeated cultures of patients with Corynebacterium amycolatum It presents data from repeated cultures with *Corynebacterium amycolatum*.

Patient ID	Isolated microorganism	Co-infecting microorganism
3	Corynebacterium amycolatum	None
4	Corynebacterium amycolatum	None
5	Corynebacterium amycolatum	None
8	Corynebacterium amycolatum	None

To clarify the inclusion criteria, patients with positive cultures for *C. amycolatum* were included regardless of the presence of other bacteria. The determination of *C. amycolatum* as the primary pathogen was based on repeated isolation and correlation with clinical symptoms, in the absence of a cytological study.

Antibiotic susceptibility testing (Table 3) was performed on the isolated *C. amycolatum* strains against the following antibiotics: levofloxacin, moxifloxacin, cefotaxime, ceftriaxone, cefepime, penicillin, ampicillin, benzylpenicillin, gentamicin, erythromycin, clindamycin, vancomycin, tetracycline, tigecycline, trimethoprim/sulfamethoxazole, and linezolid. A total of 15 susceptibility tests were conducted for levofloxacin, with 13.3% (n=2) of isolates being susceptible and 86.6% (n=13) resistant. For moxifloxacin, three tests were performed, with 33.3% (n=1) susceptible, 33.3% (n=1) intermediate, and 33.3% (n=1) resistant. Cefotaxime was tested in six cases, with 100% (n=6) susceptibility. Ceftriaxone was tested in 14 cases, showing 78.5% (n=11) susceptibility, 7.1% (n=1) intermediate, and 14.2% (n=2) resistance. Nine tests for cefepime showed 100% (n=9) susceptibility. Eight penicillin tests showed 25% (n=2) susceptibility, 37.5% (n=3) intermediate, and 37.5% (n=3) resistance. Fifteen tests were conducted for ampicillin, with 46.6% (n=7) susceptible, 20% (n=3) intermediate, and 33.3% (n=5) resistant. Four benzylpenicillin tests showed 50% (n=2) susceptibility and 50% (n=2) intermediate response. For gentamicin (n=9), 11% (n=1) were intermediate, and 89% (n=8) were resistant. Erythromycin was tested in 13 cases: 15.3% (n=2) were susceptible, 15.3% (n=2) were intermediate, and 69.2% (n=9) were resistant. Clindamycin was tested in 10 cases, showing 10% (n=1) intermediate response and 90% (n=9) resistance. Vancomycin was tested in five cases, all of which (100%, n=5) were susceptible. Tetracycline showed 100% (n=6) susceptibility in six tests. Tigecycline was tested in two cases, both showing 100% (n=2) susceptibility. Trimethoprim/sulfamethoxazole was tested in nine cases, all of which (100%, n=9) were resistant. Lastly, 15 tests were performed for linezolid, with 100% (n=15) susceptibility.

**Table 3:** Antibiotic susceptibility profile for Corynebacterium amycolatum S, susceptible; I, intermediate; R, resistant; N/A not applicable

Antibiotics	Antibiogram															
	1	2	3	4	5	6	7	8	9	10	11	12	13	14	15	16
Levofloxacin	R	R	R	R	N/A	R	R	R	S	R	R	R	R	R	S	R
Moxifloxacin	N/A	N/A	R	N/A	N/A	I	N/A	N/A	S	N/A	N/A	N/A	N/A	N/A	N/A	N/A
Cefotaxime	S	N/A	S	N/A	N/A	S	S	S	S	N/A	N/A	N/A	N/A	N/A	N/A	N/A
Ceftriaxone	S	S	S	S	N/A	S	R	S	N/A	S	S	S	S	S	R	I
Cefepime	N/A	S	N/A	S	N/A	N/A	N/A	N/A	N/A	S	S	S	S	S	S	S
Penicillin	N/A	S	N/A	R	N/A	N/A	N/A	N/A	N/A	I	S	R	I	R	N/A	I
Ampicillin	R	S	S	R	N/A	S	S	S	S	I	S	R	I	R	R	I
Benzylpenicillin	S	N/A	S	N/A	N/A	I	I	N/A	N/A	N/A	N/A	N/A	N/A	N/A	N/A	N/A
Gentamicin	N/A	R	N/A	R	N/A	N/A	N/A	N/A	N/A	I	R	R	R	R	R	R
Erythromycin	R	I	N/A	R	N/A	R	S	N/A	S	R	R	R	I	R	R	R
Clindamycin	R	N/A	R	R	N/A	R	N/A	R	N/A	N/A	R	N/A	I	R	R	R
Vancomycin	S	N/A	S	N/A	N/A	S	S	S	N/A	N/A	N/A	N/A	N/A	N/A	N/A	N/A
Tetracycline	S	N/A	S	N/A	N/A	S	S	S	S	N/A	N/A	N/A	N/A	N/A	N/A	N/A
Tigecycline	N/A	N/A	S	N/A	N/A	S	N/A	N/A	N/A	N/A	N/A	N/A	N/A	N/A	N/A	N/A
Trimethoprim/sulfamethoxazole	N/A	R	N/A	R	N/A	N/A	N/A	N/A	N/A	R	R	R	R	R	R	R
Linezolid	S	S	S	S	N/A	S	S	S	S	S	S	S	S	S	S	S

**Figure 1:**
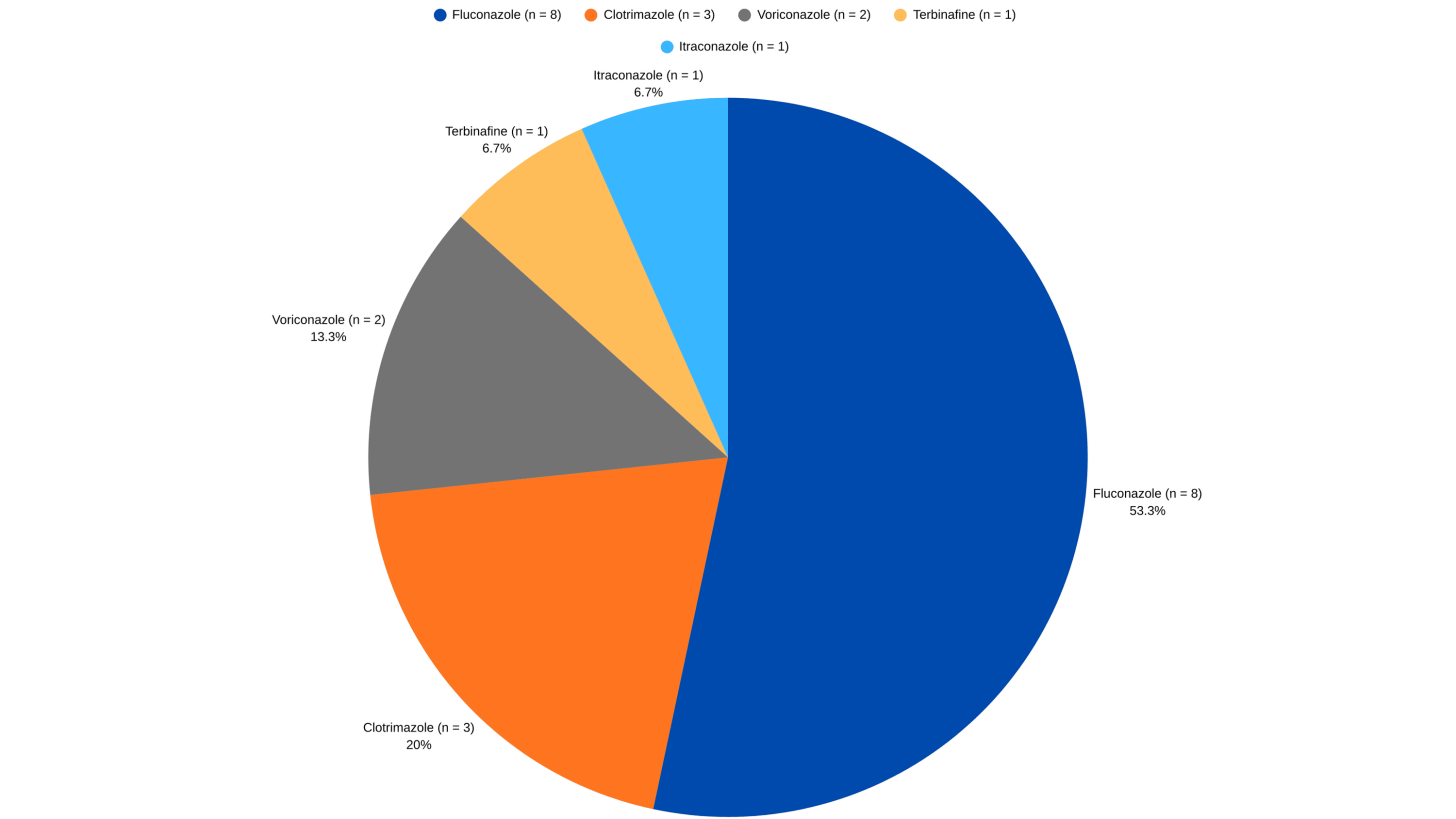
Empiric antifungal agents used in treatment Data are presented as n (%), where n represents the number of cases.

**Figure 2:**
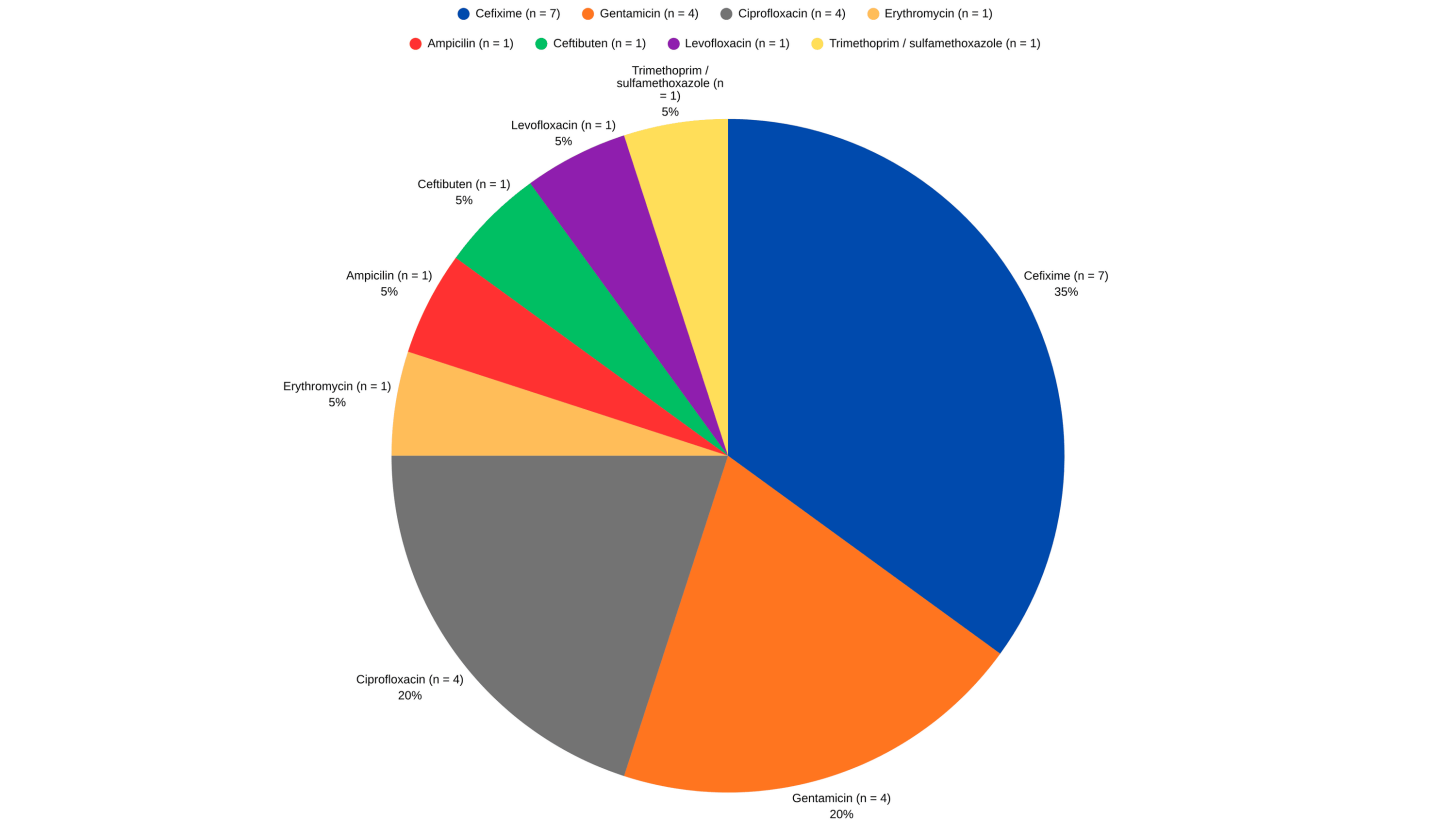
Empiric antibiotics used in treatment Data are presented as n (%), where n represents the number of cases.

Nonsurgical treatment was primarily empiric, based on a combination of antibiotic therapy, antifungal agents, and regular cleaning of the affected ear, supplemented with a drying solution or boric acid. Boric acid powder was used in 75% (n=9) of patients. The most commonly used antifungal was fluconazole, while the primary antibiotic prescribed was cefixime (Figure 1 and Figure 2). Once the causative agent was identified, treatment was administered based on the antibiotic susceptibility profile.

After the isolation of *C. amycolatum*, four patients showed clinical improvement with antibiotic therapy and did not require surgical intervention. In contrast, eight patients underwent surgical procedures due to persistent infections.

**Figure 3:**
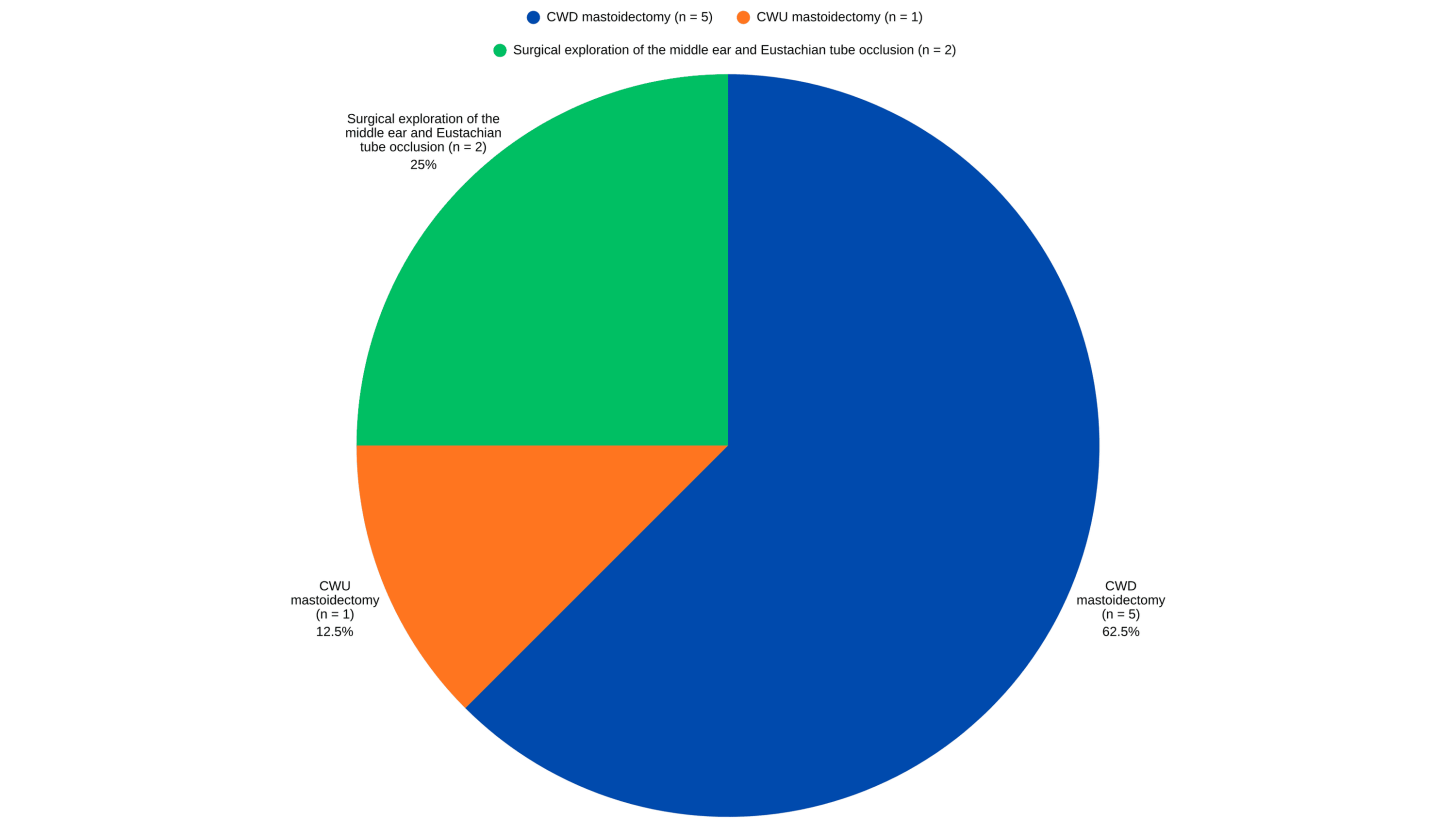
Surgical interventions in treatment CWD, canal wall-down; CWU, canal wall-up

Among the four patients who had more than one positive culture for *C. amycolatum*, 75% (n=3) required surgery. Of all patients, 62.5% (n=5) underwent canal wall-down (CWD) mastoidectomy, 25% (n=2) required surgical exploration of the affected middle ear along with Eustachian tube occlusion, and 12.5% (n=1) underwent canal wall-up (CWU) mastoidectomy (Figure 3).

During the follow-up period, no documented cases of relapse or reinfection confirmed by *C. amycolatum* were recorded in patients who underwent surgery and completed antibiotic treatment adjusted according to the antibiogram.

## Discussion

*C. amycolatum* has gained relevance as a potential contributor to COM, yet its clinical significance and resistance patterns remain insufficiently explored. Our study aimed to describe its microbiological profile and clinical associations, and the findings suggest a possible role in persistent infections, particularly in patients with cholesteatoma, while highlighting resistance patterns that may have implications for empiric therapy selection.

The susceptibility profile observed in our study is partly consistent with previous international reports, which also describe high susceptibility to vancomycin and linezolid and variable resistance to quinolones and clindamycin. Conversely, it has become susceptible to beta-lactams such as third- and fourth-generation cephalosporins and penicillins. The observed susceptibility pattern highlights how antibiotic use can lead to resistance and mutations in this bacterium [22].

From a clinical perspective, the resistance pattern observed suggests that quinolones, commonly used as first-line agents in COM, may be unreliable when *C. amycolatum* is suspected. In contrast, the preserved susceptibility to cephalosporins and penicillins indicates that these agents are more suitable options for empiric therapy in our setting. This pattern may justify reconsidering local empiric guidelines to include beta-lactams when Corynebacterium species are suspected, particularly in patients with recurrent disease or cholesteatoma.

Similar observations have been reported by Sengupta et al., who described *C. amycolatum* as an unexpected but clinically relevant pathogen in ear infections, emphasizing that it should not always be regarded as a contaminant or commensal organism [7]. This correlates with our findings, in which *C. amycolatum* was identified in patients with COM, most of whom presented with cholesteatoma and persistent disease.

According to the United States National Institutes of Health, more than 60% of infections are caused by biofilms, and approximately 60% of cholesteatomas are associated with them [15,23]. Fujikawa T demonstrated that the most common biofilm-forming agents in cholesteatoma are *Corynebacterium*, *Brevibacterium*, and *Cutibacterium*, while *Corynebacterium*, *Staphylococcus*, and *Pseudomonas* are predominant in COM [24]. Our study aligns with the literature by confirming that *C. amycolatum* is a bacterium known for its ability to form biofilms associated with cholesteatoma development.

In our study, 91% (n=11) of patients presented with cholesteatoma. Once the causative agent was identified, treatment was administered based on the isolated pathogen's antibiotic susceptibility profile, with cefixime used as the antibiotic in 58% of cases, yielding favorable outcomes. Other antibiotics used less frequently included trimethoprim/sulfamethoxazole, levofloxacin, ciprofloxacin, and erythromycin. Combination therapy with fluconazole was chosen due to the patients’ clinical characteristics, as some authors suggest that COM caused by fungi is a consequence of bacterial infection and its treatment [25].

In the analysis of the patients included in the study, comorbidities such as diabetes mellitus, hypertension, and obesity were observed. These findings highlight the importance of considering patients' overall health status when assessing the severity of the infection and deciding on the appropriate treatment.

The limitation of this study was the inability to perform molecular studies, such as polymerase chain reaction (PCR), to confirm the isolates with greater certainty. Additionally, it was not possible to carry out the same antibiotic susceptibility testing profile for all *C. amycolatum* strains. It was also not feasible to standardize a single antibiotic treatment regimen for all patients following their surgeries, as the procedures were personalized for each case. Another challenge we faced was the limited information currently reported about this pathogen and its implications in COM and cholesteatoma. Finally, the small sample size limits the generalizability of our findings and underscores the need for larger studies to validate these observations.

## Conclusions

This study highlights *C. amycolatum* as a potential contributor to COM, suggesting a possible role in persistent infections and emphasizing its notable resistance to commonly used antibiotics. These findings support the importance of considering this organism in diagnostic evaluations and adopting an updated approach to the management of COM, including targeted therapy based on culture and susceptibility testing. Given the limited global evidence and the small sample size of our study, larger studies are needed to better define the clinical significance of *C. amycolatum*, clarify its resistance mechanisms, and determine the most effective therapeutic strategies.

## References

[1] Schilder AG, Marom T, Bhutta MF (2017). Panel 7: otitis media: treatment and complications. Otolaryngol Head Neck Surg.

[2] Khairkar M, Deshmukh P, Maity H, Deotale V (2023). Chronic suppurative otitis media: a comprehensive review of epidemiology, pathogenesis, microbiology, and complications. Cureus.

[3] Gupta S, Kumbhat P (2022). Microbiology of chronic otitis media and shifting trends of its antibiotic susceptibility: a prospective observational study in a tertiary care Institute of Western Rajasthan. Indian J Otolaryngol Head Neck Surg.

[4] Esteban J, Nieto E, Calvo R, Fernández-Robals R, Valero-Guillén PL, Soriano F (1999). Microbiological characterization and clinical significance of Corynebacterium amycolatum strains. Eur J Clin Microbiol Infect Dis.

[5] Oteo J, Aracil B, Alós J (2001). Significant bacteremia caused by Corynebacterium amycolatum: an emerging pathogen. Infect Dis Clin Microbiol.

[6] Wauters G, Van Bosterhaut B, Janssens M, Verhaegen J (1998). Identification of Corynebacterium amycolatum and other nonlipophilic fermentative corynebacteria of human origin. J Clin Microbiol.

[7] Sengupta M, Naina P, Balaji V, Anandan S (2015). Corynebacterium amycolatum: an unexpected pathogen in the ear. J Clin Diagn Res.

[8] Olender A, Bogut A, Magryś A, Król-Turmińska K (2018). A novel approach to study the effect of ciprofloxacin on biofilms of Corynebacterium spp. using confocal laser scanning microscopy. Pol J Microbiol.

[9] Olender A, Bogut A, Magryś A, Tabarkiewicz J (2019). Cytokine levels in the in vitro response of T cells to planktonic and biofilm Corynebacterium amycolatum. Pol J Microbiol.

[10] Abbott P, Gunasekera H, Leach AJ (2016). A multi-centre open-label randomised non-inferiority trial comparing watchful waiting to antibiotic treatment for acute otitis media without perforation in low-risk urban Aboriginal and Torres Strait Islander children (the WATCH trial): study protocol for a randomised controlled trial. Trials.

[11] Aguilar-Morales L, Soley-Gutiérrez C, Arguedas-Mohs A. (2006). Pharmacokinetic and pharmacodynamic principles in the treatment of children with otitis media. Acta Médica Costarricense.

[12] Martins C, Faria L, Souza M (2009). Microbiological and host features associated with corynebacteriosis in cancer patients: a five-year study. Mem Inst Oswaldo Cruz.

[13] Bhumireddy SK, Gudla SS, Vadaga AK, Nandula MS (2025). Vancomycin-induced DRESS syndrome: a systematic review of case reports. Hosp Pharm.

[14] Sahu KK, Taneja J, Kumar A, Dey A (2024). Vancomycin-induced DRESS syndrome: a comprehensive review of cases and pathophysiology. J Clin Pharm Ther.

[15] Mena Viveros N (2014). Biofilms in otolaryngology. Acta Otorrinolaringol Esp.

[16] Hogan CA, Watz N, Budvytiene I, Banaei N (2019). Rapid antimicrobial susceptibility testing by VITEK®2 directly from blood cultures in patients with Gram-negative rod bacteremia. Diagn Microbiol Infect Dis.

[17] Graf B, Adam T, Zill E, Göbel UB (2000). Evaluation of the VITEK 2 system for rapid identification of yeasts and yeast-like organisms. J Clin Microbiol.

[18] Ligozzi M, Bernini C, Bonora MG, De Fatima M, Zuliani J, Fontana R (2002). Evaluation of the VITEK 2 system for identification and antimicrobial susceptibility testing of medically relevant gram-positive cocci. J Clin Microbiol.

[19] Funke G, Monnet D, deBernardis C, von Graevenitz A, Freney J (1998). Evaluation of the VITEK 2 system for rapid identification of medically relevant gram-negative rods. J Clin Microbiol.

[20] Eigner U, Schmid A, Wild U, Bertsch D, Fahr AM (2005). Analysis of the comparative workflow and performance characteristics of the VITEK 2 and Phoenix systems. J Clin Microbiol.

[21] Riccobono E, Aiezza N, Niccolai C, Giani T, Rossolini GM (2023). Evaluation of VITEK® 2 AST cards (AST-N376 and AST-N397) for susceptibility testing of challenging Gram negatives. Diagn Microbiol Infect Dis.

[22] Ozdemir S, Aydogan O, Koksal Cakirlar F (2021). Biofilm formation and antimicrobial susceptibility of non-diphtheria Corynebacterium strains isolated from blood cultures: first report from Turkey. Medeni Med J.

[23] Saunders J, Murray M, Alleman A (2011). Biofilms in chronic suppurative otitis media and cholesteatoma: scanning electron microscopy findings. Am J Otolaryngol.

[24] Fujikawa T, Tanimoto K, Kawashima Y (2022). Cholesteatoma has an altered microbiota with a higher abundance of Staphylococcus species. Laryngoscope Investig Otolaryngol.

[25] Levi J. (2026). Chronic suppurative otitis media (CSOM): clinical features and diagnosis. https://www.uptodate.com.

